# Prerequisites for Clinical Implementation of Whole‐Heart 4D‐Flow MRI: A Delphi Analysis

**DOI:** 10.1002/jmri.29550

**Published:** 2024-08-21

**Authors:** Joost van Schuppen, Annelies E. van der Hulst, J. Michiel den Harder, Lukas M Gottwald, Raschel D. van Luijk, Josien C. van den Noort, Jules L. Nelissen, Casper F. Coerkamp, S. Matthijs Boekholdt, Paul F.C. Groot, Aart Nederveen, Pim van Ooij, R. Nils Planken, Delphi Panel Members

**Affiliations:** ^1^ Department of Radiology and Nuclear Medicine Amsterdam UMC, University of Amsterdam The Netherlands; ^2^ Atherosclerosis & Ischemic Syndromes Amsterdam Cardiovascular Sciences Amsterdam The Netherlands; ^3^ Emma Children's Hospital, Department of Pediatric Cardiology Amsterdam UMC, University of Amsterdam Amsterdam The Netherlands; ^4^ Medical Imaging Quantification Centre Amsterdam UMC, Academic Medical Center Amsterdam The Netherlands; ^5^ Amsterdam Movement Sciences Amsterdam The Netherlands; ^6^ Department of Cardiology Amsterdam UMC, University of Amsterdam Amsterdam The Netherlands; ^7^ Present address: Philips Healthcare Best The Netherlands

**Keywords:** Delphi technique, 4D‐flow MRI, cardiovascular disease

## Abstract

Whole‐heart 4D‐flow MRI is a valuable tool for advanced visualization and quantification of blood flow in cardiovascular imaging. Despite advantages over 2D‐phase‐contrast flow, clinical implementation remains only partially exploited due to many hurdles in all steps, from image acquisition, reconstruction, postprocessing and analysis, clinical embedment, reporting, legislation, and regulation to data storage. The intent of this manuscript was 1) to evaluate the extent of clinical implementation of whole‐heart 4D‐flow MRI, 2) to identify hurdles hampering clinical implementation, and 3) to reach consensus on requirements for clinical implementation of whole‐heart 4D‐flow MRI. This study is based on Delphi analysis. This study involves a panel of 18 experts in the field on whole‐heart 4D‐flow MRI. The experience with and opinions of experts (mean 13 years of experience, interquartile range 6) in the field were aggregated. This study showed that among experts in the cardiovascular field, whole‐heart 4D‐flow MRI is currently used for both clinical and research purposes. Overall, the panelists agreed that major hurdles currently hamper implementation and utilization. The sequence‐specific hurdles identified were long scan time and lack of standardization. Further hurdles included cumbersome and time‐consuming segmentation and postprocessing. The study concludes that implementation of whole‐heart 4D‐flow MRI in clinical routine is feasible, but the implementation process is complex and requires a dedicated, multidisciplinary team. A predefined plan, including risk assessment and technique validation, is essential. The reported consensus statements may guide further tool development and facilitate broader implementation and clinical use.

**Level of Evidence:**

NA

**Technical Efficacy:**

Stage 5

Whole‐heart 4D‐flow MRI (three‐dimensional time‐resolved phase‐contrast MRI) is an imaging technique that enables advanced visualization and quantification of blood flow.^1^, ^2^, ^3^ It allows simultaneous quantification of flow in any location within the acquired volume of interest.^1^, ^2^, ^3^ This eliminates variations between multiple consecutive 2D‐phase‐contrast (PC) acquisitions and can also reduce scan time.^1^, ^2^, ^3^ At least similar reliability of whole‐heart 4D flow as compared to conventional 2D PC MRI has been reported and whole‐heart 4D‐flow MRI has proven to be of additional value in congenital heart disease (CHD) and valvular heart disease (VHD).^1^, ^2^, ^3^, ^4^, ^5^, ^6^, ^7^, ^8^, ^9^ However, clinical implementation of whole‐heart 4D‐flow MRI remains only partially exploited in current clinical care.

Specific technical requirements and clinical indications for whole‐heart 4D‐flow MRI have been comprehensively described.^1^, ^3^ Nevertheless, the clinical implementation of whole‐heart 4D‐flow MRI remains impeded by hurdles in all steps needed, from image acquisition, reconstruction, postprocessing and analysis, clinical embedment of 4D‐flow MRI, reporting, legislation, and regulation to data storage. Overcoming these hurdles requires specific knowledge and expertise. Currently, in order to clinically implement 4D‐flow MRI, every center individually encounters these hurdles without guidance on how to overcome them. Accordingly, the analysis in this study attempts to cluster expert knowledge on hurdles and solutions for the clinical implementation of whole‐heart 4D‐flow MRI.

The Delphi method is a systematic process that may be used to structure expert knowledge and opinions.^10^, ^11^, ^12^ By iteration, a Delphi study allows for nuancing and reconsideration of the experts' opinions based on the anonymized opinions of others. Currently, Delphi studies are well‐established in formulating consensus statements in the medical field.^10^, ^11^, ^12^

The aim of this paper is to 1) investigate the extent of clinical implementation of whole‐heart 4D‐flow MRI; 2) identify major hurdles that hamper clinical implementation of whole‐heart 4D‐flow MRI by using a Delphi analysis; and 3) reach a consensus on what is needed for clinical implementation of whole‐heart 4D‐flow MRI. Ultimately, the outcomes may facilitate widespread clinical implementation of whole‐heart 4D‐flow MRI by guiding new and current users to address the major hurdles that currently hamper clinical implementation.

## Materials and Methods

A conventional, three‐round Delphi method was utilized to address all challenges and hurdles experienced with whole‐heart 4D‐flow MRI, including solutions found.^11^, ^12^ In addition, a questionnaire was conducted to determine the current experience and opinions of the Delphi panelists on whole‐heart 4D‐flow MRI in clinical care.

### Topics and Questions

**Figure 1:**
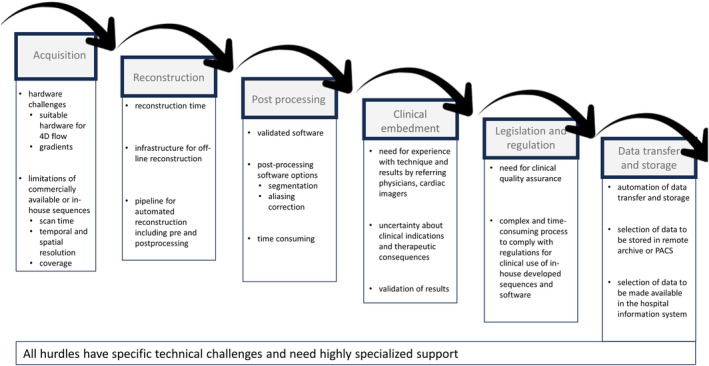
Topics of whole‐heart 4D‐flow MRI.

The topics of the Delphi analysis were determined by a structured approach, aiming to identify all possible hurdles and experience on clinical implementation of whole‐heart 4D‐flow MRI. Topics were at first identified based on in‐house experiences before and after implementing whole‐heart 4D‐flow MRI in clinical practice, as described in the following text (Fig. 1). These topics were compared with a recent consensus statement, and adjusted where needed.^3^, ^13^ Delphi panelists were allowed to suggest additional topics for any missing topics.

Topics were categorized following the clinical workup of whole‐heart 4D‐flow MRI, from acquisition to discussing results with clinicians and patients (Table 1):

- Technical aspects of whole‐heart 4D‐flow acquisition
- Clinical implementation and indications
- Reconstruction of the whole‐heart 4D‐flow data and postprocessing
- Integration of whole‐heart 4D‐flow MRI data in the clinical report
- Embedment of whole‐heart 4D‐flow MRI results in clinical decision‐making
- Legislation and regulation
- Storage of whole‐heart 4D‐flow MRI datasets

**Table 1:** Topics for Delphi Analysis

1. Technical aspects of whole‐heart 4D‐flow acquisition	Feasibility of clinical implementation Technical aspects and specifications of whole‐heart 4D‐flow acquisition
2. Clinical implementation and indications	Practically embedding 4D‐flow acquisition in a clinical MRI protocol Clinical indications
3. Reconstruction of the whole‐heart 4D‐flow data and postprocessing of this data	Features and specifications essential for clinical implementation Specifications of segmentation and quantification Validation of results
4. Integration of whole‐heart 4D‐flow MRI data in the clinical report	Reporting of whole‐heart 4D‐flow results Essential components of a report
5. Embedment of whole‐heart 4D‐flow MRI results in clinical decision‐making	Arguments for clinical implementation of whole‐heart 4D‐flow MRI Responsibility for validity and quantification Value of advanced whole‐heart 4D‐flow MRI parameters
6. Legislation and Regulation	Influence of legislation and regulation on clinical implementation of whole‐heart 4D‐flow MRI
7. Storage of the whole‐heart 4D‐flow MRI datasets	Storage of the (typically) large whole‐heart 4D‐flow MRI datasets

Open questions were composed, addressing these topics. These questions were evaluated on content and readability by a focus group, consisting of nine experts on imaging, technique, or 4D‐flow MRI. This group is separate from the group of Delphi panelists, including two imagers (14 and 10 years of experience), three engineers (24, 24, and 14 years of experience), one referring physicians (4 years of experience), two clinical scientists (experience 20 and 16 years), and one researchers in the field (8 years of experience) (Appendices S2 and S3).

### Panel Members

For the Delphi analysis, experts in the field were recruited, based on their experience with whole‐heart 4D‐flow MRI in either research or clinical setting. Delphi panelists were selected to represent the full spectrum of expertise with whole‐heart 4D‐flow MRI, from centers with no clinical implementation in place, to centers having whole‐heart 4D‐flow MRI fully implemented. Twenty‐two potential panelists were invited from academic medical centers in North America, Europe, and Asia. The selection criteria to be invited were experience with the use of 4D flow, either in research or in clinical setting. We attempted to include an equal share of panelists with either clinical or scientific experience, as well as different levels of implementation. The group of panelists together published 68 articles in 2023, of a total number 208 published in 2023 (Appendix A). Nineteen members accepted the invitation. Of these 19 panel members, 17 completed the survey, and one partially completed the survey of the first round, but participated in rounds 2 and 3. The answers of this participant to the open questions in round 1 were used to formulate statements for round 2 and kept in the analysis. One member did not complete the survey. The 18 members had a median experience of 13 years and interquartile range 6 years. Panel member details are listed in Table 2.

**Table 2:** Panel Members

Country of residence/Practice	Canada	1
	France	2
	Egypt	1
	South Korea	1
	The Netherlands	2
	United Kingdom	2
	United States of America	6
	Sweden	1
	Switzerland	2
Background	Clinical	11/18
	Technical	7/18

Research and Clinical Experience With 4D‐Flow MRI	Median	Interquartile Range
Years of experience with 4D‐flow MRI	13	6
Annual 4D‐flow MRI research examinations (scans)	100	147
Total published research paper per institution of panelists	25	53
Annual 4D‐flow MRI (scans) in clinical care patients	108	200

After an introduction meeting, explaining the methods and timeline, the online questionnaire was distributed to the panelists using Castor EDC.^14^

After finalizing the Delphi analysis a feedback meeting was organized, where results were presented and discussed.

### Delphi Analysis

#### ROUND 1

The first Delphi round consisted of a questionnaire composed of two sections. The first section was designed to obtain a comprehensive overview of the panel members regarding their clinical and scientific experience with and opinions on whole‐heart 4D‐flow MRI, and the extent of clinical implementation of whole‐heart 4D‐flow MRI in the centers of the panelists (Appendix S1). The second section offered a set of 59 in‐depth open questions regarding the previously identified topics (Appendix S2). In the last part of this section, the experts were invited to provide suggestions for additional topics that were not addressed in the set of 59 questions.

#### ROUND 2

From the answers to the open questions in the first round and the additional suggestions from the panelists, 34 statements were formulated encompassing the full clinical patient journey (three statements on technical challenges of whole‐heart 4D‐flow acquisition, seven on practically embedding a whole‐heart 4D‐flow sequence in a clinical MRI protocol, nine on the reconstruction of the whole‐heart 4D‐flow data and postprocessing of this data, six on the clinical embedment of whole‐heart 4D‐flow MRI, five on the integration of results of whole‐heart 4D‐flow MRI in the report, two on the legislation and regulation issues with the application of 4D‐flow MRI, and two on the data storage of the large sets typically used in whole‐heart 4D‐flow MRI). In the event of different responses to open questions, provided that there were detailed descriptions of specific elements within the workflow or technique, these descriptions were consolidated into a single statement. These distinct elements were separately evaluated for their relevance and appropriateness as part of the workflow or technique. Appendix S2 describes answers to open questions, from which statements were composed.

These statements were presented to the panel members in round 2. Panel members were asked to rate the statements using a 3‐point Likert scale (disagree, neutral, or agree). Free comments were allowed after rating each statement, which aided in rephrasing those statements not reaching consensus. Consensus was defined as 75% of panel members showing agreement with regard to the Likert score. Statements that reached consensus were not presented in the third round. Appendix S3 describes the results of rating of the statements formulated in rounds 2 and 3.

#### ROUND 3

Statements that did not reach consensus in the second round were rephrased, taking into account the issues raised by the panel members. In the third round, the rephrased statements were again presented to the panel members and scored using the same Likert scale. All statements that reached consensus (in round 2 or 3) are presented in the results section of this article. Statements that did not reach consensus are shown in Appendix S3 and elaborated on in the discussion.

## Results

### Delphi Analysis

Round 1, section 1: Questionnaire on panelist's opinion and experience with whole‐heart 4D‐flow MRI.

#### TECHNICAL ASPECTS OF WHOLE‐HEART 4D‐FLOW ACQUISITION

MRI facilities and resources of panelists institutions are summarized in Table 3.

**Table 3:** Four‐Dimensional‐Flow MRI Sequences Used in Institution of Panelists

	Description	*N*
Vendor supplied	Cartesian sequence, with conventional reconstruction (Siemens)	3
	Cartesian sequence, with compressed sensing reconstruction/WIP (Siemens)	6
	Cartesian sequence, with Kat‐ARC reconstruction (kat ARC = 8), 20 msec, 2 × 2 × 2 mm (GE)	2
	Cartesian gradient‐echo sequence, retrospective cardiac gating, and respiratory navigator gating (Phillips)	1
	Radial slice to volume flow sensitive phase contrast (investigational)	1
	Not further specified/“vendor supplied”	5
	Retrospective True FISP sequence, 30 phases, 2 × 2 × 2 mm/3 × 3 × 3 mm. Free breathing (Siemens)	2
	(Adaptation of) Cartesian compressed sensing under sampling, 40 msec, 2 × 2 × 2 mm ECG triggered (Phillips)	1
	EPI sequence, EPI factor 5–7	1
In house developed	Compressed sensing sequence	1
	PC VIPR: radially under‐sampled 4D‐flow sequence	1
	Self‐developed sequence (TFE) 1	1
Field strength	1.5–3.0 T	
Vendor	Siemens, Phillips, GE	
Availability of sequence on scanner	Readily, without extra action	12/13
	Only available after extra action (patch or reboot)	1/13

WIP = work in progress; Kat‐ARC = k‐adaptive‐t autocalibrating reconstruction for Cartesian sampling; VIPR = vastly undersampled isotropic projection; EPI = echo‐planar imaging; TFE = turbo field echo; FISP = fast imaging with steady‐state precession.

The panel consisted of members who use vendor‐provided sequences as well as those who use in‐house developed sequences for whole‐heart 4D‐flow MRI. Of the sequences used, 83% meet the requirements for clinical use as described in the consensus paper.^3^ The 17% of sequences that did not fulfill these requirements, concerned longer scanning time and or a lack of spatial and temporal resolution. Panel members used various solutions to accelerate the 4D‐flow acquisition, the most common solution was a smaller coverage/volume of interest. Also, reduced spatial resolution and a higher acceleration factor are reported.

#### RECONSTRUCTION OF THE WHOLE‐HEART 4D‐FLOW DATA AND POSTPROCESSING

The need for—and use of—several solutions to accelerate image reconstruction of whole‐heart 4D‐flow MRI were reported by the panelists. Solutions found included upgrading hardware capacity and performance, utilization of acceleration techniques (examples given included compressed sensing [CS] factors 5–10 and parallel imaging [eg, SENSE or GRAPPA factor 2]), and specific vendor‐supplied techniques (Appendix S1). However, not all members described a need to reduce reconstruction time (five described no special need for a solution, eight found a specific solution, and five lacked a specific solution). Details on response regarding reconstruction are summarized in Table 4. The response regarding the process of postprocessing and quantification, including software is summarized in Table 5.

**Table 4:** Details of Reconstruction of 4D‐Flow Sequences Used in Institution of Panel Members

Location of reconstruction	MRI machine	15/18
	Off‐line server	1/18
	Both	2/18
Software used for reconstruction of 4D‐flow sequence		
In‐house developed software		9/18
Commercially available software	Vendor specific	17/18
	MEDIS software	
	Mass research	
	MATLAB	
	PIE medical	
	Arterys	
	Circle *Cvi42*®	
	GTI flow	
	Ensight	
Use of both in‐house developed and commercially available software		10/18
Research tool (not approved for clinical care)		1/18

In most institutions, reconstruction includes preprocessing, such as background correction and aliasing correction. In 7/18 institutions (part of) preprocessing is done with a separate tool, separate from the process of reconstruction.

**Table 5:** Solutions for Postprocessing and Quantification of 4D‐Flow Data Used in Institution of Panel Members

Performed by	Cardiac imager	13/17
	Trained technician	7/17
	Researcher	13/17
Software used for postprocessing and quantifications	Circle *Cvi42*®	
	MEDIS suite MR 4D flow	
	Mass research	
	Arterys	
	MATLAB Circle	
	Gyroflow	
	Ensight 4D flow	
	PIE Medical CAAS	
	GTI flow	
	Ensight	
	In‐house developed software	

#### INTEGRATION OF WHOLE‐HEART 4D‐FLOW MRI DATA IN THE CLINICAL REPORT AND EMBEDMENT OF WHOLE‐HEART 4D‐FLOW MRI RESULTS IN CLINICAL DECISION‐MAKING

Successful clinical implementation of whole‐heart 4D‐flow MRI was reported by 68% of panel members. Reasons for unsuccessful clinical implementation included limitations regarding acquisition time, insufficient hardware/software, or a lack of indications or institutional approval, summarized in Table 6. Panelists reported various indications for clinical whole‐heart 4D‐flow MRI (Table 7).

**Table 6:** Clinical Implementation of 4D‐Flow MRI in Institution of Panel Members

Clinically Implemented	13/18 (68%)	
Reasons not to implement^5^	Lack of time for post processing	3
	Lack of validation	1
	No time in clinical slots for 4D flow	1
	Lack of indication	1
	Lack of an available sequence on the scanner	1
	Lack of approval or priority of the institution	2

**Table 7:** Clinical Indications for Implementation of Whole‐Heart 4D‐Flow MRI According to Panelists That Implemented 4D‐Flow MRI

Standard care in all cardiovascular patients	3/13
Analysis of congenital heart disease for diagnosis	8/13
Follow‐up of congenital heart disease	8/13
Valvular heart disease	7/13
Suspicion of cardiac shunt	5/13
Congenital disease of the aorta	8/13
Acquired disease of the aorta	5/13

### Delphi Rounds

After round 2, consensus was reached for 29 (85%) statements. Five statements (15%) (S1.1, S1.3, S2.6, S2.7, and S5.6) needed rephrasing, taking into account all comments of the panelists, and were presented in round 3 (Appendix S3). After rephrasing, in round 3, all 34 statements reached consensus.

### CONSENSUS STATEMENTS

1. Technical aspects of whole‐heart 4D‐flow acquisition
  1.1 Clinical implementation of 4D‐flow MRI for basic cardiovascular application is feasible. For whole‐heart 4D‐flow MRI in complex cases, such as CHD, technical challenges, lack of validated and standardized sequences as well as postprocessing tools are still hampering clinical implementation.
  1.2 Despite the long scanning time for most sequences, current commercially available 4D‐flow sequences meet the minimal clinical requirements for basic indications. For more complex pathology, there is a need for more advanced options of faster acquisition.
  1.3 The use of Conformité Européenne (CE) approved tools for 4D‐flow MRI is recommended. However, when such a tool is not available or does not meet the clinical requirements, the use of in‐house developed software for acquisition and reconstruction is considered a good alternative for clinical practice, provided full technical support is available, including (software code) maintenance.
2. Clinical implementation and indications
  2.1 Under ideal circumstances, a 4D‐flow sequence with postprocessing and quantification methods should be standard care in cardiovascular imaging.
  2.2 With the current state‐of‐the‐art 4D‐flow sequence with postprocessing and quantification methods, whole‐heart 4D‐flow MRI should be used for specific indications and should not be considered a routine sequence in a standard cardiac MRI examination.
  2.3 Clinical indications for whole‐heart 4D‐flow MRI*:
    - CHD (for diagnosis prior to intervention or surgery) (100%)
    - VHD (100%)
    - Follow‐up of CHD after correction (94%)
    - Suspicion of cardiac shunt (89%)
    - Acquired disease of the aorta, for example, aneurysm, dissection (89%)
    - Congenital disease of the aorta, for example, coarctation aortae, connective tissue disease, Marfan's disease, Loeys Dietz (78%)
  2.4 Specifications of an accelerated 4D‐flow acquisition, essential for clinical implementation*:
    - A simple predefined whole‐heart sequence, easily plannable and tunable (89%)
    - High resolution/accuracy (72%)
    - Acquisition time <5 minutes (39%)
    - Self‐gating sequence, for respiratory and cardiac motion (39%)
    - A sequence without the need for IV contrast (39%)
    - Dual VENC (22%)
  2.5 For clinical care, a whole‐heart 4D‐flow MRI sequence should be standardized, with limited features adaptable, such as size, VENC, acceleration factor, and spatial and temporal resolution.
  2.6 When flow is assumed to be within normal velocity range a standard VENC per indication should be used, and a VENC scout is not required. In suspicion of abnormal flow velocity and absence of prior information such as ultrasonography data on flow velocity, a scout 2D‐PC can be helpful for estimation of the optimal VENC setting. A phase unwrapping algorithm should be available to solve aliasing.
  2.7 For accurate planning of a whole‐heart 4D‐flow MRI sequence a localizer in three orientations, as included in the standard MRI protocol, is sufficient.
3. Reconstruction of the whole‐heart 4D‐flow data and postprocessing
  3.1 Features and specification of an accelerated whole‐heart 4D‐flow MRI reconstruction essential for clinical implementation*:
    - Reconstruction on MRI console without interference with subsequent acquisition (78%)
    - Reconstruction time <5 minutes, immediately available for quality assurance/VENC adjustment (72%)
    - Correction integrated in reconstruction (61%)
    - Integrated pipeline without human interference. Data sent back after reconstruction to MRI console (56%)
    - Applied artificial intelligence (AI) (correction, reconstruction) for facilitated and accelerated use (33%)
    - Reconstruction location remote (server/cloud/off line) (17%)
  3.2 Features and specifications of whole‐heart 4D‐flow MRI pre‐ and postprocessing pipeline essential for clinical implementation*:
    - Easily accessible options for quality assurance (83%)
    - Adequate Picture archiving and communication system (PACS) interaction (78%)
    - Pipeline, parallel or in line, with results within 10 minutes (67%)
    - Production of optimized data for further analysis and visualization (61%)
    - Automated data handing, back to MRI console/operator console for quality assessment, postprocessing, quantification, and interpretation (55%)
    - AI‐based automation of segmentation, background phase correction, plane selection, flow analysis, and volumetry for facilitated and accelerated use (44%)
  3.3 The acceptable time spent on postprocessing and quantification in routine whole‐heart 4D‐flow MRI is up to 15–20 minutes per case. For more complex cases this might be more.
  3.4 For commercially available whole‐heart 4D‐flow sequences, vendors should provide a reconstruction pipeline on the MRI scanner, for standardized clinical care.
  3.5 Ideally, postprocessing and quantification of whole‐heart 4D‐flow MRI are performed in the reading room, on the PACS station where interpretation and reporting are performed.
  3.6 Segmentation of whole‐heart 4D‐flow MRI data should be fully automated, with easy options for manual corrections. With the current state of technology, semiautomated segmentation would suffice.
  3.7 In standard cases, postprocessing for visualization and quantification in whole‐heart 4D‐flow MRI can be performed by a well‐trained, experienced technician, supervised by a cardiac imager. For complex cases, such as CHD both visualization and quantification are part of understanding anatomy and physiology and therefore should be interpreted and authorized by the cardiac imager.
  3.8 4D‐flow MRI results should be validated, ideally in all scans. Validation can be performed using additional 2D‐PC, conservation of mass, or volumetric data.
  3.9 Technical support for 4D‐flow MRI scanning should be readily available, but not necessary on site.
4. Integration of whole‐heart 4D‐flow MRI data in the clinical report
  4.1 There is a need for formulating a reporting standard for whole‐heart 4D‐flow MRI, similar to 2D‐PC. Institutional and international standards might be useful.
  4.2 In the reporting of whole‐heart 4D‐flow MRI the following components are important*:
    - Conventional flow parameters (net and forward flow, regurgitant flow/fraction, peak velocities) (100%)
    - Shunt measurements (94%)
    - Key images of findings (94%)
    - Volumetry (end‐diastolic volume [EDV], EDV/body surface area [EDV/BSA], end‐systolic volume [ESV], stroke volume [SV], ejection fraction [EF]) (89%)
    - Reference values (when available) (89%)
    - Written report with qualitative interpretation of the findings (83%)
    - Validation of results (with addition 2D‐PC or conservation of mass) (83%)
    - Flow curves (visual) (61%)
    - Qualitative visualization of flow (streamlines, time resolved path lines, flow paths, flow velocities vectors) (56%)
  4.3 Visualization of results provides a qualitative insight into hemodynamics and abnormalities thereof and is helpful in the communication of findings and understanding of whole‐heart 4D‐flow MRI results to noncardiac imagers. These should allow for direct comparison with other imaging modalities, such as echocardiography.
  4.4 When whole‐heart 4D‐flow MRI data is sufficiently validated and clinically integrated, the use of 2D‐PC results for internal validation can be omitted from the report. In case of discrepancies, they should be included.
  4.5 Uncertainties in 4D‐flow MRI data should be expressed and explained in the report.
5. Embedment of whole‐heart 4D‐flow MRI results in clinical decision‐making
  5.1 Arguments for clinical implementation for whole‐heart 4D‐flow MRI in daily clinical practice are:
    - Efficiency, including less scan time (planning and acquisition), no need for repeating scans (94%)
    - Postprocessing capabilities, including visualization of flow/velocities, multivalvular analysis (89%)
    - Easy plannable, operator‐independent acquisition (89%)
    - Standardization, higher accuracy (78%)
    - New insights into disease with advanced parameters (78%)
    - Patient comfort (67%)
  5.2 When transitioning from 2D‐PC to 4D‐flow MRI, ideally the whole team should be involved. The person in the lead, preferably a cardiac imager, should have expertise in the 4D‐flow MRI technique.
  5.3 After implementation and validation of whole‐heart 4D‐flow MRI in clinical practice, 2D‐PC can be omitted, in most cases. In specific cases, such as for VENC estimation or in case of expected high peak velocity, the use of 2D‐PC can still be of additional value.
  5.4 The diagnostic value and clinical importance of advanced whole‐heart 4D‐flow MRI parameters still have to be proven before integration as a biomarker in clinical care is feasible.
  5.5 The following advanced whole‐heart 4D‐flow MRI parameters are expected to become of clinical importance*:
    - Visualization of flow (89%)
    - Wall shear stress (56%)
    - Flow displacement (42%)
    - Surrogates for pressure such as turbulent kinetic energy, pressure maps, energy loss (39%)
    - Markers of flow eccentricity (29%)
    - Flow components (28%)
  5.6 If while awaiting CE‐certified commercially available whole‐heart 4D‐flow MRI, an in‐house developed solution is used, the responsibility for the validity and quantification of whole‐heart 4D‐flow MRI data lies with the multidisciplinary in‐house team, including the cardiac imager
6. Legislation and regulation
  6.1 Legislation and regulation help create awareness for robust testing and validation before clinical use and integration of whole‐heart 4D‐flow MRI. Robust testing and validation improve safety and help adoption and integration of techniques in clinical use. However, in practice this might slow down the process of implementation and integration.
  6.2 Legislation and regulation influence the choice of hardware and software for whole‐heart 4D‐flow MRI. CE approved soft‐ and hardware will be chosen above nonapproved soft‐ and hardware.
7. Data storage
  7.1 The original phase‐contrast reconstructed whole‐heart 4D‐flow MRI datasets need to be archived for long time storage, similar to medical images, given that this is part of the patient's record. This might allow future advanced flow parameter analysis as well.
  7.2 Reconstructed DICOM images should ideally be stored in PACS. Off‐line storage might be an option as well, to avoid the need for large storage capacity in PACS.

*Percentage between brackets represents percentage of panelists that scored or agreed with item on relevance or importance.

## Discussion

### Delphi Results/Statements

This Delphi analysis shows that, although whole‐heart 4D‐flow MRI is currently used for clinical and research purposes among experts in the cardiovascular field, experts agree that major hurdles hamper broad clinical implementation and utilization. The present article provides consensus statements that may be used as a guide in tool development and broader clinical implementation of whole‐heart 4D‐flow MRI.

The expert panel agrees that the currently commercially available whole‐heart 4D‐flow sequences meet the minimal clinical requirements for standard basic clinical applications, such as (acquired) VHD, suspicion of shunts, and aortic disease. However, the panel clearly points out that several technical challenges are still hampering clinical implementation in more complex cases, specifically in CHD, where patients would benefit most from the advantages of whole‐heart 4D‐flow MRI implementation. Clear advantages of 4D‐flow MRI over conventional 2D PC flow sequences, as reported by the panelists, include efficiency, less scanning time, easier planning, and operator‐independent acquisition with less need for repetition of scans. In addition, multivalvular analysis as well as advanced imaging biomarkers, including flow patterns (direction, distribution, vorticity, helicity, disturbance), energy (kinetic energy, viscous energy loss), and component flow analysis^15^, are important arguments for standard implementation of 4D‐flow MRI in the clinical workflow.

Regarding the technical challenges, the panel indicates a clear need for the availability of a standardized sequence with only limited features adaptable, such as scanning range, VENC, acceleration factor, and spatiotemporal resolution.

Required improvements in the currently available sequences include shorter scan time and less dependence on experience with the specific sequence. Especially with the continuous demand for shorter scan slots, less than 30 minutes, scantimes down to 5 minutes are of importance. Furthermore, there is a need for solutions for time‐consuming segmentation and postprocessing of data for efficient clinical application of 4D‐flow MRI in the broad setting of clinical cardiovascular care.

The reported statements were presented and discussed during an online feedback session with the Delphi panelists. Regarding a few statements, some important comments should be acknowledged. Statements 3.1 and 3.2, on the utilization of artificial intelligence lack precise definition and description. However, according to the panel, there is a high anticipation for the future application of artificial intelligence in tasks such as reconstruction, segmentation, and postprocessing. In specific cases, this could enhance efficiency and reduce the complexity experienced by using current postprocessing tools in whole‐heart 4D‐flow MRI.

In statement 2.4, dual VENC is proposed as an essential component of an accelerated 4D‐flow acquisition for clinical implementation. It was initially anticipated to score a high consensus rate, yet this statement only achieved a low score During the feedback session it became clear that this low score might be attributed to a too narrow definition. This statement should have encompassed a broader range of solutions for a dynamic range problem, such as antialiasing and dual VENC.

### Why Is Clinical Implementation of Whole‐Heart 4D‐Flow MRI Important?

In the ideal setting of clinically implemented whole‐heart 4D‐flow MRI, flow data are acquired in one volume of interest, encompassing the entire heart and proximal part of the great arteries in a single scan Blood flow can afterwards be quantified in any plane or subvolume within this volume of interest. A single whole‐heart 4D‐flow acquisition can replace multiple 2D‐flow acquisitions resulting in a substantial reduction in time required for (multiple) 2D planning and acquisition. It substantially reduces the number of breath‐holds and total scan time, making it more time‐efficient and more tolerable for patients.^3^, ^6^, ^7^, ^9^ Whole‐heart 4D‐flow MRI thereby offers easier scan execution with fewer planning errors and avoid the interacquisition variation that between 2D‐PC acquisitions. The ability to quantify flow in any desired plane within the volume of interest, including the simultaneous quantification of flow across all four valves, makes the acquisition less operator‐dependent, eliminating human errors in flow quantification caused by suboptimal plane positioning and angulation.^3^, ^6^, ^9^ Additionally, whole‐heart 4D‐flow MRI has much more to offer than substituting 2D flow because it allows for quantification of advanced imaging biomarkers; flow patterns (direction, distribution, vorticity, helicity, disturbance), energy (kinetic energy, viscous energy loss), and component flow analysis.^15^

Furthermore, apart from technical hurdlers, there is a human factor required for successful implementation of whole‐heart 4D‐flow MRI in a broad clinical setting. For positioning in clinical decision‐making, the advantages of the technique need to be clear not only to the cardiac imagers but also to the referring physician and the patient. Currently, echocardiography and 2D‐PC MRI hold their strong position because cardiac imagers and treating clinicians are not yet sufficiently familiar with whole‐heart 4D‐flow MRI. Only when the benefits of 4D‐flow MRI compared to 2D flow MRI, in terms of accurate diagnosis and clinical decision‐making, are clear to the entire team will there be an incentive to utilize this technique in clinical practice.

### Future Perspective

The results of our Delphi analysis provide a detailed overview of all aspects needing improvement for broad implementation and utilization of whole‐heart 4D‐flow MRI. This study may be helpful for colleagues who aim for clinical utilization of this technique. We expect that our analysis may be used as a guide to improvement of 4D‐flow acquisition, reconstruction, and postprocessing software facilitating embedment in clinical care and decision‐making. Our results can further be used as recommendations towards vendors on issues the field would like to see improved. Also, the consensus statements in this paper can be used for further development of statements and guidelines on clinical use of 4D flow, to provide more practical solutions for daily use.

### Limitations of this Study

Delphi studies have several limitations.^11^, ^16^ A bias is introduced by the group that composes the questions. To avoid this, the questions were reviewed by a feedback group, not only consisting of imagers, but also clinicians, physicists, physicians, and researchers. Furthermore, in round 1 all panel members were given the opportunity to comment on topics that needed to be addressed or were missing according to their opinion.

In addition, it is inevitable in a Delphi analysis that the of the selection of panel member may have introduced possible bias despite our effort to select representatives with different levels of experience and clinical implementation worldwide.

## Conclusion

This Delphi analysis shows that, among experts in the cardiovascular field, whole‐heart 4D‐flow MRI is currently used for both clinical and research purposes, with some experts using commercially available tools, while others rely on in‐house developed tools, partially due to vendor specific constraints.

Overall, the panelists agree that major hurdles currently hamper smooth implementation and utilization of this technique.

The reported consensus statements may guide further tool development and facilitate broader implementation and clinical use.

## Supporting information


**Appendix S1** Questions and answers on experience and opinions on clinical use 4D‐flow MRI by panelists.

**Appendix S2** Questions answers by panelists in round 1 Delphi analysis with resulting statements for round 2.

**Appendix S3** Result of panelists rating of statements in rounds 2 and 3.

## Appendix A Collaborators

Alex J. Barker, PhD; Malenka Bissell, MD, PhD; Petter Dyverfeldt, PhD; Christopher Francois, MD; Pankaj Garg, MD, PhD; Julio Garcia, PhD; Julia Geiger, MD; Rajesh Krishnamurthy, MD; Tim Leiner, MD, PhD; Michael Markl, PhD; Jean‐François Paul, MD; Eva S. Peper, PhD; Soha Romeih, MD, PhD, FESC, FSCMR; Eric M. Schrauben, PhD; Jean‐Michel Serfaty, MD, PhD; Jos J.M. Westenberg, MD, PhD; Oliver Wieben, PhD; Dong Hyun Yang, MD, PhD.

## Appendix B Delphi Panel Members

- Alex J. Barker, PhD, Department of Radiology, Children's Hospital Colorado, University of Colorado Anschutz Medical Center, Aurora, USA
- Malenka Bissell, MD, PhD, Department of Biomedical Imaging Science, Leeds Institute of Cardiovascular and Metabolic Medicine (LICAMM), LIGHT Laboratories, Clarendon Way, University of Leeds, Leeds, LS2 9NL, UK
- Petter Dyverfeldt, PhD, Department of Health, Medicine and Caring Sciences, Linköping University, Linköping, Sweden; Center for Medical Image Science and Visualization, Linköping University, Linköping, Sweden
- Christopher Francois, MD, Cardiovascular and Thoracic Divisions, Mayo Clinic, 200 First St SW, Rochester, MN 55905, USA
- Pankaj Garg, MD, PhD, Department of Cardiovascular and Metabolic Health, Norwich Medical School, University of East Anglia, Norwich, Norfolk, United Kingdom
- Julio Garcia, PhD, Departments of Radiology and Cardiac Sciences, University of Calgary, Alberta, Canada
- Julia Geiger, MD, Department of Diagnostic Imaging, University Children's Hospital, Zurich, Switzerland
- Rajesh Krishnamurthy, MD, Department of Radiology, Nationwide Children's Hospital, Columbus, OH 43205, USA
- Tim Leiner, MD, PhD, University Medical Center Utrecht, Department of Radiology, Utrecht, the Netherlands; Mayo Clinic, Department of Radiology, Rochester, Minnesota, USA
- Michael Markl, PhD, Department of Radiology, Feinberg School of Medicine, Northwestern University, Chicago, IL, USA
- Jean‐François Paul, MD, Institut Mutualiste Montsouris, Department of Radiology, Cardiac Imaging, 75014 Paris, France
- Eva S. Peper, PhD, Department of Diagnostic, Interventional and Pediatric Radiology (DIPR), Inselspital, Bern University Hospital, University of Bern, Bern, Switzerland
- Soha Romeih, MD, PhD, Radiology Department, Aswan Heart Centre, Aswan, Egypt; Cardiology Department, Tanta, Egypt
- Eric M. Schrauben, PhD, Department of Radiology & Nuclear Medicine, Amsterdam University Medical Centers, Amsterdam, Netherlands
- Jean‐Michel Serfaty, MD, PhD, Department of Diagnostic Cardio‐Vascular Imaging, CHU de Nantes, Nantes Université, l'institut du thorax, CNRS, INSERM 1087, France
- Jos J.M. Westenberg, MD, PhD, CardioVascular Imaging Group (CVIG), Department of Radiology, Leiden University Medical Center, Leiden, The Netherlands
- Oliver Wieben, PhD, Departments of Medical Physics, Radiology, University of Wisconsin, Madison, WI, USA
- Dong Hyun Yang, MD, PhD, Department of Radiology, Research Institute of Radiology, Asan Medical Center, University of Ulsan College of Medicine, Seoul, Korea
